# Assessment of the phytochemical, antioxidant and antibacterial activities of *Heteromorpha arborescens *(Spreng.) Cham & Schltdl. leaf extracts

**DOI:** 10.12688/f1000research.25197.1

**Published:** 2020-09-01

**Authors:** Taiwo Oluwafunmilola Abifarin, Gloria Aderonke Otunola, Anthony Jide Afolayan

**Affiliations:** 1 Medicinal Plants and Economic Development (MPED) Research Centre, Department of Botany, University of Fort Hare, Alice 5700, Eastern Cape, South Africa

**Keywords:** Phytochemicals, Heteromorpha arborescens, antioxidant, antibacterial, bioactives, blanching, traditional, medicines.

## Abstract

**Background:** *Heteromorpha arborescens *(Spreng.) Cham. and Schltdl (Apiaceae) is widely used traditionally for the treatment of a wide range of diseases in Southern and Eastern Africa. Although previous studies have reported the biological activities of hexane, ethyl acetate and methanol extracts of *H. arborescens* leaves, there is no scientific information on the phytochemical contents, antioxidant and antibacterial activities of acetone, ethanol, aqueous and blanched extracts. This study is therefore aimed to investigate and compare the phytochemical contents, antioxidant and antibacterial activities of acetone, ethanol, aqueous and blanched extracts of *H. arborescens* leaves.

**Methods**: Phytochemical analysis for the total phenolic, flavonoid, proanthocyanidin, alkaloid and saponin contents of all the fractions were determined by spectroscopic methods, while the free radical scavenging potential of the extracts were evaluated using DPPH, ABTS radical scavenging and total antioxidant capacity assays. Micro dilution method was used to determine the Minimum Inhibitory Concentrations (MIC) of *H. arborescens* leaf extracts against *Bacillus pumilus, Staphylococcus epidermidis, Staphylococcus aureus, Escherichia coli* and *Klebsiella pneumoniae*.

**Results**: Total phenol content of the extracts ranged between 15.10 mg GAE/g- 42.50 mg GAE/g, proanthocyanidin was 459-8402.1 mg QE/g, and flavonoid content of 109.24-235.79 mg QE/g. In addition, alkaloids (5.59%) and saponins (23.33%) were present in significant amounts. Based on the IC _50_ values, the ethanol extract exhibited the highest total antioxidant activity (0.013 mg/mL) with highest inhibition against DPPH and ABTS radicals (0.06 and 0.049 mg/mL respectively). Considerable antibacterial activities were observed in the acetone, ethanol and blanched extracts with MIC values ranging from 1.563-12.5 mg/mL; however, the aqueous extract was inactive against all the bacteria strains.

**Conclusion**: The study suggests that *H. arborescens* leaves could be a valuable source of bioactive compounds. Although the blanching process significantly decreased polyphenolic contents and antioxidant activities of the extracts, it increased the antibacterial compounds.

## Introduction

Plants from the Apiaceae family are generally known to be rich sources of phytochemicals and antioxidants and are commonly used as food, flavoring agents and medicine ^1^. These bioactive constituents may contribute significantly to the protection of humans from a wide range of diseases ^2– 4^. *Heteromorpha arborescens* (Spreng.) Cham. and Schltdl otherwise referred to as parsley tree in English, “wildepitersielie” in Afrikaans and “umbangandlala” in Xhosa is a large shrub or small tree that belongs to the Apiaceae family ^3^.

The leaves vary from simple to compound and the flowers are small, greenish to yellowish in colour, occurring in compound umbels, with waxy bark which is smooth or glossy in texture ^5^. The species is generally considered as a medicinally important plant throughout Africa since almost all its parts are traditionally used for the treatment of different ailments ^6^. In South Africa, the leaves and roots are used for blood purification, diabetes and shortness of breath ^7– 9^. The bark, leaves and roots are also used for respiratory problems in Kenya, Lesotho and Tanzania ^9^. The leaves are consumed as vegetables in Kenya ^10^, while the roots are given to malnourished children in Botswana and Swaziland ^11^. The plant is also used to treat abdominal pains, dysmenorrhea, nervous and mental disorders, as well as a vermicide in children ^12^.

The healing ability of the plant is attributed to the presence of bioactive compounds, such as tannins, phenols, alkaloids, saponin, flavonoids, and proanthocyanidin. Although very scanty, previous scientific reports exist on the phenolic contents, antioxidant ^13, 14^ and antibacterial activities ^3^ of hexane, ethyl acetate and methanol extracts of *H. arborescens* leaves. To the best of our knowledge no study exists on comparison of polyphenolic contents, antioxidant and antibacterial activities of blanched, aqueous, acetone and ethanol extracts of *H. arborescens* leaves. The present study was therefore conducted to determine the phytochemical contents, antioxidant and antibacterial activities of blanched, aqueous, acetone and ethanol extracts of *H. arborescens* leaves.

## Methods

### Ethical approval

Ethical approval was granted by the University of Fort Hare Animal and Plant Use Research Ethics Committee, South Africa with protocol number OTA011SABI01/19/E.

### Chemical and reagents

All reagents and chemicals including gallic acid, rutin, quercetin, aluminum chloride (AlCl _3_), ferric chloride (FeCl _3_.7H _2_0), Folin- Ciocalteu, oxalic acid, trichloroacetic acid (TCA), sulphuric acid (H _2_SO _4_), hydrochloric acid (HCl), Na _2_CO _3_, ammonium molybdate, potassium ferricyanide (K _3_Fe(CN) _6_), catechin, 2,2-diphenyl-1-picrylhydrazyl (DPPH) and 2,2’-azinobis (3-ethylobenzothiazoline-6-sulphonic acid diammonium salt) (ABTS) are products of Sigma-Aldrich, South Africa. Other chemicals such as anhydrous sodium carbonate, sodium nitrite (NaNO _2_), ethanol, methanol, n-butanol, sodium acetate, butylated hydroxytoluene (BHT), diethyl ether, glacial acetic acid, sulfanilic acid, potassium persulphate, sodium nitroprusside were also purchased from the same company. All the reagents used were of analytical grade. The bacterial strains including; *Klebsiella pneumoniae* ATCC 13883, *Staphylococcus aureus*, ATCC 29213, *Pseudomonas aeruginosa* ATCC 19582, *Escherichia coli* 25922, *Bacillus pumilus* ATCC 14884, *Staphylococcus epidermis* ATCC 12228 were obtained from the Department of Biochemistry and Microbiology, University of Fort Hare, South Africa.

### Plant material

Fresh leaves of *H. arborescens* were obtained in the month of June, 2019 from an area located on latitude 32° 47′ 50.4″ S, 26° 52′ 41.8″ E along Hogsback road, Alice Town, Eastern Cape, South Africa. The plant was authenticated by a taxonomist at the University of Fort Hare and a voucher specimen was preserved in the Giffen Herbarium and assigned number Abif2019/03.

### Preparation of plant extracts

Fresh *H. arborescens* leaves were oven dried at 40 ^0^C and crushed to coarse powder. 100 g of the powdered plant material was extracted separately in 500 mL acetone, ethanol and water for 24 hours on an orbital shaker. An equal weight of the fresh leaves was immersed into 500 mL of hot water (80 ^0^C) for 5 min to simulate home cooking and ground with a blender. The extracts were filtered under pressure using a Buchner funnel and Whatman filter paper (150 mm); the acetone and ethanol extracts were concentrated to dryness using a rotary evaporator while the aqueous extract and blanched sample were subjected to freeze drying. The extracts were thereafter stored at 4 ^0^C until further analysis.

### Phytochemical screening

***Determination of total phenolic content.*** The total phenolic content was determined by spectrometry using Folin-Ciocalteu reagent. Briefly, 0.5 mL of extract (1mg/mL) and gallic acid (0.02 to 0.1mg/mL) were put separately in test tubes. Thereafter, 2.5mL of 10% Folin-Ciocalteu reagent was added into the test tubes, after which 2.5 mL of 7.5% sodium carbonate solution was added to the mixture, stirred and incubated at 40 ^0^C for 30 min. The absorbance was then measured at 760 nm using a Hewlett Packard VR-2000 spectrophotometer. All samples were analysed in triplicate. Total phenolic content was expressed as milligrams of gallic acid equivalent per gram (mg GAE/g) with the standard curve: y =10.875 x+ 0.1025, R ^2 ^= 0.996.

Where R is the determined coefficient, x is the concentration, and y is the absorbance. 

***Determination of flavonoids content.*** The amount of flavonoids present in the extract was determined using the aluminium chloride (AlCl _3_) colorimetric assay, as described by Majouli *et al.* ^15^. A mixture of 0.5 mL of extract was added to an equal volume of 2% AlCl _3_ solution. The mixture was incubated for 10 min and vigorously shaken, after which the absorbance was measured at 420 nm. Varying concentrations of the standard (quercetin) were also prepared with the same method. All samples were analysed in triplicates. Flavonoid content was expressed as milligrams of quercetin equivalent per gram (mg QE/g) with the standard curve: y = 2.9422x - 0.4438, R ^2^ = 0.913

Where R is the determined coefficient, x is the concentration, and y is the absorbance.

***Determination of proanthocyanidin (condensed tannins).*** Proanthocyanidin content was determined as described by Sagbo *et al.* ^16^. Briefly, a mixture of 3mL of 4% vanillin-methanol solution and 1.5mL of HCl was added to 0.5 mL of each extract. The solution was stirred and incubated at 27 ^∘^C for 15 min, after which absorbance was measured at 500 nm. All samples were analysed in triplicate and the amount of proanthocyanidin was expressed as mg/g dry weight of quercetin equivalent (mg QE/g) of the extract with the standard curve: y = 0.0252 x + 0.0482, R ^2^ = 0.9005.

Where R is the determined coefficient, x is the concentration, and y is the absorbance.

***Determination of alkaloid content.*** Alkaloid content of *H. arborescens* leaves was determined as previously described by Abifarin *et al*. ^17^. 0.5 g of the pulverized leaves was mixed with 200mL of 10% acetic acid in ethanol. The mixture was covered, incubated at room temperature for 4 h, filtered and concentrated to about a quarter of its original volume in a water bath. To the extract, concentrated ammonium hydroxide was added in drops till precipitation was complete. After the solution was allowed to settle, precipitates obtained were washed with dilute ammonium hydroxide and then filtered. The residue was dried in an oven (40°C), weighed and the alkaloid content was determined using the following formula:

% Alkaloid = weight of precipitate / initial weight of sample × 100.

***Determination of saponin content.*** Saponin content of *H. arborescens* leaves was determined as previously described by Unuofin *et al.* ^18^. Briefly, 0.5 g of pulverized *H. arborescens* leaves was measured into 50 mL of 20% ethanol prepared in distilled water. The mixture was heated in a hot water bath for 4 h at 55°C. The mixture was filtered and the residue extracted again with another 50 mL of 20% ethanol. The two filtrates were combined and reduced to 20 mL over a hot water bath (90°C). The concentrated solution obtained was poured into a 250 mL separating funnel containing 20 mL of diethyl ether. The aqueous layer was collected while the ether layer was discarded. 20 mL of n-butanol was added to the filtrate and then washed thrice with 10 mL of 5% sodium chloride. The mixture was heated in an oven (40°C) to constant weight. The percentage saponin content of the sample was calculated using the following formula:

% Saponins = weight of final filtrate / weight of sample x 100

### Antioxidant activity

***DPPH radical scavenging activity.*** DPPH radical scavenging activity for each plant extract was determined as previously described by Ohikhena *et al*. ^19^. Briefly, a reaction mixture containing 2.5 mL of DPPH solution (0.13 mM) and 2.5 mL of each plant extract or standard (rutin and BHT) dissolved in methanol at varying concentrations (0.005, 0.01, 0.02, 0.04, 0.08 mg/mL), stirred and kept in the dark for 30 min. The absorbance was measured at 517 nm and DPPH radical scavenging activity was calculated as:

% DPPH scavenging activity = [(Abs DPPH − Abs Sample) Abs DPPH)] × 100

Where Abs control is the absorbance of DPPH radical + methanol; Abs sample is the absorbance of DPPH radical + sample extract/standard.

***ABTS radical scavenging assay.*** ABTS scavenging activity of the different plant extracts was determined as described by Unuofin *et al.* ^20^. A mixture was prepared by reacting 7 mM ABTS solution and 2.45 mM K _2_S _2_O8 (1:1), which was kept in the dark for 12 hours to produce a bluish green coloration. The solution was adjusted with methanol until an absorbance of 0.700 ± 0.01 at 734 nm was obtained. 1mL of plant extract was allowed to react with 1 mL of the working solution and the absorbance was measured at 734 nm after 7 min. The ABTS scavenging capacity of the extracts were compared with that of BHT and rutin and percentage inhibition was calculated as:

ABTS radical scavenging activity (%) = [(Abs control − Abs sample) / (Abs control)] × 100.

Where Abs control is the absorbance of ABTS radical + methanol; Abs sample is the absorbance of ABTS radical + sample extract/standard.

***Total antioxidant capacity (phosphomolybdenum) assay.*** The total antioxidant capacity was determined by the method described by Abifarin *et al.* ^17^. Briefly, 0.3 mL of the extracts and standard (0.025-0.4 mg/mL) were measured into separate test tubes and each was dissolved in 3 mL of reagent solution (0.6 M sulfuric acid, 4 mM ammonium molybdate, and 28 mM sodium phosphate). The test tubes were incubated at 90°C in a water bath for 90 min, allowed to cool to room temperature and the absorbance was measured at 695 nm. Rutin and BHT were used as standards. The percentage inhibition (%TAC) was calculated as:

%TAC = [(Absorbance of sample − Absorbance of control) / (absorbance of sample)] × 100

### Determination of antibacterial activity

***Microorganisms and media.*** The bacteria used in this study were chosen primarily on the basis of their importance as opportunistic microorganisms for humans with diabetes mellitus. The bacterial strains include: *Klebsiella pneumoniae* ATCC 13883, *Staphylococcus aureus*, ATCC 29213, *Pseudomonas aeruginosa* ATCC 19582, *Escherichia coli* 25922, *Bacillus pumilus* ATCC 14884, *Staphylococcus epidermidis* ATCC 12228.

***Minimum Inhibitory Concentration (MIC).*** The MIC of the extracts were evaluated against selected Gram positive ( *Bacillus pumilus, Staphylococcus aureus* and *Staphylococcus epidermidis*) and Gram negative bacteria ( *Klebsiella pneumoniae, Pseudomonas aeruginosa* and *Escherichia coli*) by microdilution method, as described by Mohsenipour *et al*. ^21^. The bacterial concentration in the inoculum was standardized at 0.5 McFarland turbidity scale, (1 × 10 ^8^ CFU/ml).

Firstly, sterile round bottom 96-well plates were filled with 100 µl of distilled water. 100 µl stock fractions of 50 mg/mL of plant extract and standard (Erythromycin) were then added into the first row. The fractions were serially diluted to make varying concentrations of the plant extracts (0.78125–12.5 mg/mL) and standard (0.0625–1 µg/mL) and then 50 µl of inoculums was added into the wells. Thereafter, 100 µl sterile nutrient broth culture medium plus 50 µl of the culture of each organism was added into each well and the inoculated micro plates were incubated at 37°C for 24 h. 1% DMSO was used as the negative control while 100 µl of inoculums only was used as the growth hormone. The plates were incubated for 24 h at 37˚C, and subsequently 40 µl of 0.4 mg/mL INT dye was added to each well. The plates were gently agitated and incubated for another 30 min. The MICs were then determined as the lowest concentrations at which there was no indication of colour change to pink (no bacterial growth). 

### Statistical analyses

All data were expressed as mean ± standard deviation (SD) of triplicates and subjected to one-way analysis of variance (ANOVA). Where the data showed significant difference (p < 0.05) among the extracts, a mean separation was done using Fischer’s Least Significant Difference. MINITAB 17 statistical package was used for analysis.

## Results

### Phytochemical contents

The total phenol, proanthocyanidin and flavonoid contents of *H. arborescens* leaf extracts are presented in Table 1. The highest phenol content was found in the ethanol extract (42.50 mg GAE/g), followed by the aqueous extract (34.24 mg GAE/g), blanched extract (21.42 mg GAE/g) and acetone extract (15.10 mg GAE/g). Ethanol extract (8402.1 mg QE/g) showed the highest proanthocyanidin content followed by acetone extract (4923 mg QE/g DW), blanched extract (928.6 mg QE/g) and aqueous extract (459 mg QE/g). The concentration of flavonoid was highest in the ethanol extract (235.79 mg QE/g), followed by acetone extract (230.52 mg QE/g), aqueous extract (154.95 mg QE/g) and blanched extract (109.24 mg QE/g). Quantitative determination of the alkaloid and saponin contents of *H. arborescens* leaves also revealed low alkaloid content (5.59%) with considerable amount of saponin (23.33%).

**Table 1.** Total phenol, proanthocyanidin, flavonoid, alkaloids and saponin contents of *H. arborescens* leaves.

Extracts	Phenol (mg GAE/g)	Proanthocyanidins (mg QE/g)	Flavonoids (mg QE/g)	Alkaloids (%)	Saponins (%)
Aqueous	34.24±0.46 ^a^	459±49.9 ^a^	154.95±9.99 ^a^		
Acetone	15.10±1.38 ^b^	4923±579 ^b^	230.52±0.59 ^b^		
Blanched	21.42±0.28 ^c^	928.6±90.9 ^c^	109.24±0.17 ^c^		
Ethanol	42.50±0.69 ^d^	8402.1±89.5 ^d^	235.79±4.52 ^d^		
Pulverized	*	*	*	5.59±0.45	23.33±2.3

The results are expressed as mean ± standard deviation (n=3). Values with different superscripts are significantly different (P < 0.05) across the different extracts. *- not determined.

### Antioxidant activities

**Figure 1.**
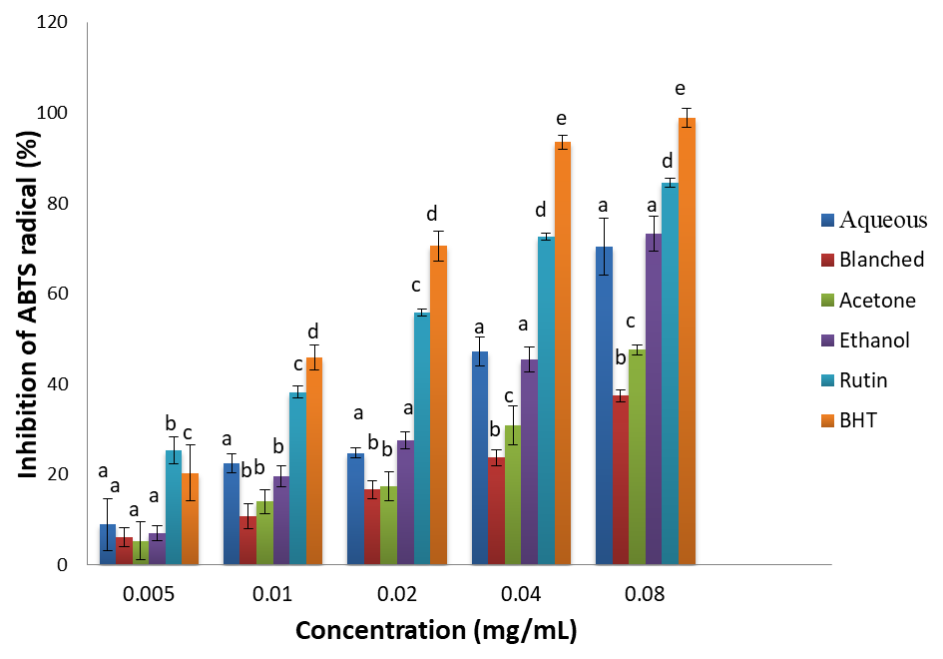
ABTS radical scavenging activity of extracts of *H. arborescens* leaf extracts and standards (rutin and BHT). Results are expressed as mean ± standard deviation (n=3). Columns with different letters are significantly different (P < 0.05) across the different samples.

***ABTS radical scavenging activity.*** The ABTS radical scavenging activities of the extracts and standards (BHT and rutin) are presented in Figure 1. The samples exhibited significant ABTS radical scavenging activities, which increased in a concentration dependent manner. Although the standards showed higher ABTS inhibitory potentials than the extracts, the highest inhibitory capacity of the extracts was observed in the ethanol and aqueous extracts. Based on the IC _50_ ( Table 2), ABTS scavenging activity of the samples was in the order: BHT > rutin > ethanol & aqueous > acetone > blanched.

**Table 2.** IC _50_ values of various solvent extracts of *H. arborescens* leaf extracts and standards.

Extracts/standard	DPPH (mg/mL)	ABTS (mg/mL)	TAC (mg/mL)
Aqueous	0.1	0.049	0.024
Blanched	0.12	0.109	0.152
Acetone	0.21	0.081	0.046
Ethanol	0.06	0.049	0.013
Rutin	0.026	0.024	0.017
BHT	0.031	0.012	0.012

**Figure 2.**
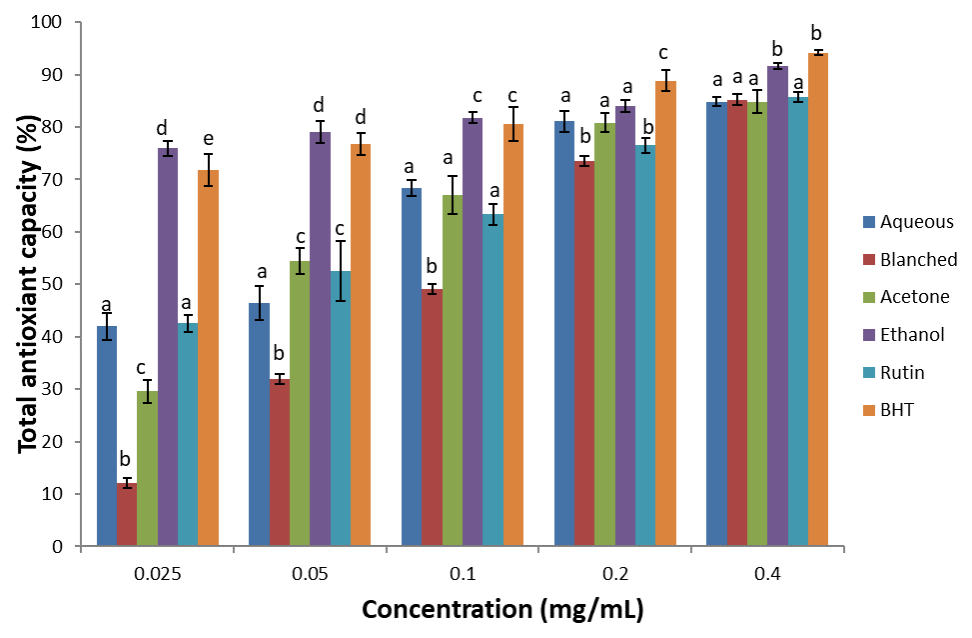
Total antioxidant capacity of *H. arborescens* leaf extracts and standards (Rutin and BHT). Results are expressed as mean ± standard deviation (n=3). Columns with different letters are significantly different (P < 0.05) across the different samples.

***Total antioxidant capacity.*** The total antioxidant capacity of the extracts and standards (BHT and rutin) are shown in Figure 2. There was a dose dependent increase in total antioxidant capacity of the samples, with the ethanol extract showing the highest antioxidant capacity when compared with other extracts. Based on the IC _50_, ( Table 2), the values of the total antioxidant capacity of the standards and extracts were in the order: BHT > ethanol > rutin > aqueous > acetone > blanched.

**Figure 3.**
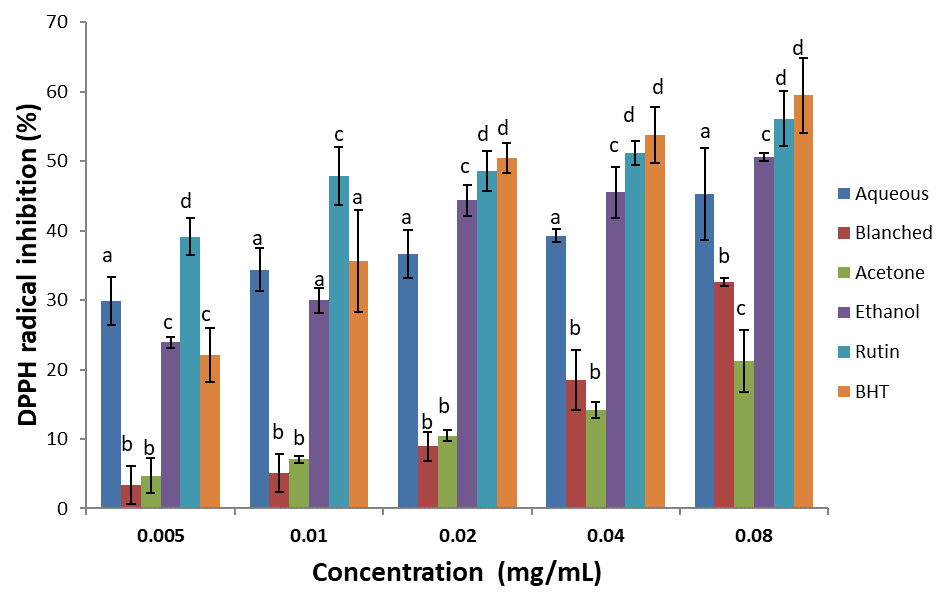
DPPH scavenging activity of *H. arborescens* leaf extracts and standards (Rutin and BHT). Results are expressed as mean ± standard deviation (n=3). Columns with different letters are significantly different (P < 0.05) across the different samples.

***DPPH scavenging activity.*** There was a dose-dependent increase in percentage inhibitory activity of the extracts and standards against DPPH radical ( Figure 3). Ethanol extracts showed the highest DPPH radical inhibitory activity; even though the standards inhibited best when compared with the extracts. With respect to the IC _50_, ( Table 2) the inhibitory capacity of the standards and extracts were in the order: rutin > BHT > ethanol > aqueous > blanched > acetone.

***Minimum inhibitory concentration.*** Antibacterial activity of erythromycin (standard) and extracts of *H. arborescens* leaves are presented in Table 3. While all Gram-positive and Gram-negative bacteria strains tested were resistant to the aqueous extract at all the concentrations tested, the blanched, acetone, and ethanol extracts showed considerable antibacterial activities with MICs ranging from 1.563-12.5 mg/mL. Ethanol extract showed the best antibacterial activity against *B. pumilus, S. epidermidis, S. aureus* and *P. aeruginosa*, exhibiting a low MIC of (1.563 and 3.125 mg/mL). Generally speaking, the Gram-negative bacteria were observed to be more resistant against the extracts tested.

**Table 3.** Minimum Inhibitory Concentration (MIC) values of *H. arborescens* leaf extracts against selected bacterial strains.

Samples	Bacterial strains (MIC) mg/mL					
	*B. pumilus ^a^*	*S. epidermidis ^a^*	*S. aureus ^a^*	*E.coli ^b^*	*K. pneumoniae ^b^*	*P. aeruginosa ^b^*
Aqueous	**Na**	**Na**	**Na**	**Na**	**Na**	**Na**
Blanched	12.5	6.25	12.5	12.5	12.5	12.5
Acetone	6.25	1.563	3.125	12.5	6.25	6.25
Etdanol	3.125	3.125	1.563	6.25	6.25	12.5
Erythromycin (µg/mL)	0.125	0.0625	0.0625	0.0625	0.125	0.125

*Bacillus pumilus, Staphylococcus epidermidis, Staphylococcus aureus, Escherichia coli, Klebselia pneumoniae.*

Bacteria ^a^- Gram positive. Bacteria ^b^- Gram negative. Na- not active at tde highest concentration tested *.*

## Discussion

Plant extracts have been reported to have numerous protective roles due to their phytochemical contents, which contributes to a large extent towards their antioxidant and antimicrobial activities ^22^. Oxidative stress, which leads to the production of free radicals in the body, plays a major role in the development of chronic and degenerative ailments such as cancer, arthritis, aging, autoimmune disorders, cardiovascular and neurodegenerative diseases ^23^. Antioxidants play a vital role in deactivating and scavenging of the free radicals in order to reduce the risk of these chronic diseases ^24^.

In the present study, the phytochemical, antioxidant and antibacterial activities of the acetone, ethanol, aqueous and blanched extracts of *H. arborescens* leaves were assessed. Phytochemical constituents varied significantly in all the extracts, which revealed the presence of phenols, flavonoids and proanthocyanidin in considerable amounts. The variation in the phytochemical constituents could be attributed to differences in the extracting capabilities of different solvents. Phenols, a major phytochemical found in plants have been widely studied because of their ability to reduce oxidative stress-related degenerative diseases, including cancer. Flavonoids are a group of hydroxylated phenolics, and their beneficial effects are linked to their antioxidant, antibacterial, anticancer, anti-inflammatory ^25, 26^ anti-hypertensive, and cardio protective activities ^27, 28^. Proanthocyanidins are a class of compounds belonging to the flavonoid family, with protective effects against tissue damage and cancer, and they also improve blood circulation by strengthening the capillaries, arteries and veins ^29^.

Ethanol has been reported to be suitable for extraction of compounds with a wide range of polarity while water is suitable for very polar compounds ^30^. Consequently, a higher quantity of phenolic compounds, proanthocyanidin and flavonoids were observed in the ethanol samples when compared to the acetone, blanched and aqueous extracts; hence, better activity. This is in agreement with previous reports that ethanol is more suitable for the extraction of phenolic compounds in plants ^30– 32^. Furthermore, high phenolic compounds were indicated in ethyl acetate and methanol extracts, suggesting the reason for its high antioxidant activity ^13^. Flavonoid content of acetone extract of *H. arborescens* was comparable to results obtained by Elisha *et al*. ^13^; however, total phenolic content was much higher as opposed to the current study.

Our results indicate that blanching resulted in a significant decrease in total phenols and flavonoids with significant increase in proanthocyanidin content. This correlates with similar observations by Korus *et al* ^33^. Jaiswal *et al* ^34^. and Irondi *et al.* ^35^. Losses of polyphenolic compounds upon blanching was also observed in some cruciferous vegetables, such as spinach ^36^, kale, broccoli ^37^ and cauliflower ^38, 39^. The decrease in polyphenolic contents could be attributed to the loss of water soluble vitamins and nitrogen compounds during the blanching process ^34^. Contrary to the findings of this study, some researchers have claimed that the heating process may alter the cell membrane, causing a release of some membrane-bound phytochemicals which may increase bioavailability as observed by Jimenez-Monreal *et al* ^40^. Similarly, Ma *et al* ^41^. observed an increase in polyphenolic contents of *Daucus carota* L. juice after blanching (at 95 ^0 ^C for 3 min).

Saponins possess medicinal properties such as anti-inflammatory, antibacterial, anticancer and cytotoxic activities ^42^. Alkaloids have also been shown to exhibit antioxidant properties by reducing oxidative damage induced by hydrogen peroxide ^43^. The amount of alkaloids and saponin exhibited by *H. arborescens* leaves is promising in playing the aforementioned biological roles.

Further assessment of the antioxidant activities of the extracts revealed the free radical scavenging potential of the extracts, which varied among the methods used. This variation was due to the fact that antioxidants are able to neutralize free radicals by different modes of action such as transition metal chelation, singlet oxygen quenchers, and donation of hydrogen ^44^.

The total antioxidant capacity and scavenging activity of the extracts against DPPH+ and ABTS+ were compared to the standards (BHT and rutin) and the extracts showed considerable antioxidant activity. Since positive correlation between polyphenolic compounds and antioxidant activities have been reported ^43, 45, 46^; the highest antioxidant activity exhibited by the ethanol extract could be attributed to its high polyphenolic compounds, as shown in this study. In terms of the relatively higher DPPH and ABTS inhibitory potential of the ethanol extract, these results are in agreement with antioxidant activity of other Apiaceae species such as: *Coriandrum sativum* ^47^, *Ammi majus* L. ^48^, *Daucus carota* L. ^49^, *Ferula gummosa* ^50^, *Seseli libanotis* (L.) Koch ^51^, *Foeniculum vulgare* and *Anethum graveolens* ^1^. This strengthens the suggestion that the most important bioactive potentials are observed in the plant extracts with high amounts of phenolics and flavonoids. This finding therefore provides some scientific verification for the biological activities of *H. arborescens* leaves. 

In recent years, more attention has been given to the development of antibacterial drugs from medicinal plants instead of synthetic ones. The antibacterial activities of plant extracts have been confirmed against an array of both Gram-positive and Gram-negative bacteria ^52^. Previous studies have established that the crude, hexane, ethyl acetate, chloroform and butanol extracts reveal antibacterial activities against *E. coli*, *S. aureus* and *P. aeruginosa*. However, *P. aeruginosa* was more susceptible to three out of the extracts with the butanol fraction showing the highest antibacterial activity ^53^. The resistance of all the bacterial strains to the aqueous extract at all concentrations tested and the significant antibacterial activity of the blanched extract obtained in the present study, could be attributed to poor solubility of the antibacterial compounds in cold water and high extractive power of boiled water. In addition, the higher antibacterial activity observed in the acetone and ethanol extracts could be due to relatively higher flavonoid and phenolic contents present ^54^.

Our findings are in agreement with McGaw *et al* ^55^. who reported that in the evaluation of antibacterial activities of hexane, ethanol and aqueous leaf extracts of *H. arborescens* against *Bacillus subtilis, S. aureus, E. coli,* and *K. pneumoniae*, only ethanol extract exhibited antibacterial activities. These findings correlate with observation made by Wigmore *et al* ^56^. that aqueous extracts of plants showed less antibacterial activity when compared with other solvents. Furthermore, in agreement with our findings, the aqueous extract was inactive against all Gram-positive bacteria used. However, contrarily, the aqueous extract was active against *S. aureus* and *S. epidermidis* ^3^. 

## Conclusion

Our results clearly indicated that the extraction of phenolic compounds and their antioxidant capacity is highly dependent on the solvent of extraction. The study also revealed that *H. arborescens* aqueous, blanched and ethanol leaf extracts possess various levels and concentrations of phytochemical constituents, which may be essential for human health. A positive correlation between polyphenolic contents, antioxidant and antibacterial activities in extracts of *H. arborescens* leaves was established, which indicates that certain phenolic compounds may be responsible for high antioxidant activity. It was also observed that the blanching process (at 80 ^0 ^C) significantly decreased polyphenolic content and antioxidant activities but increased antibacterial activity of *H. arborescens* leaves. This study therefore agrees with its reported use in traditional medicine for the treatment of some bacterial infections and diseases.

## Data availability

### Underlying data

Figshare: Absorbance values for antioxidant activity.xlsx, https://doi.org/10.6084/m9.figshare.12654479.v1 ^57^.

This project contains the following underlying data:

- Replicate absorbance values for DPPH, TAC and ABTS
- Replicate phytochemical analysis results
- MIC values of antibacterial activity of the extracts

Data are available under the terms of the Creative Commons Zero "No rights reserved" data waiver (CC0 1.0 Public domain dedication).
