# Supplementary material for: Assessment of the phytochemical, antioxidant and antibacterial activities of Heteromorpha arborescens (Spreng.) Cham & Schltdl. leaf extracts
Source: F1000Res. 2020 Sep 1;9:1079. [Version 1] doi: 10.12688/f1000research.25197.1 (PMC7533734; doi:10.12688/f1000research.25197.1)
Supplement: Peer review history

# Peer review: Reviewer response for version 1

**DOI:** 10.5256/f1000research.27806.r71376

**Published:** 2020-10-02

**Authors:** Balasubramani Ravindran

**Affiliations:** 1 Department of Environmental Energy and Engineering, Kyonggi University, Suwon, South Korea

- **Title: **The title reflects sufficiently, the work done and reported by the authors.
- **Abstract:** Adequate
- **Introduction: **This is adequate with current and relevant citations.
- **Methods:** Methods used were sufficient and reproducible by others.
- **Results** were well presented with Tables and Figures where appropriate. These were also properly captioned and can stand alone.
- **Discussion** projected the results and made appropriate reference to similar works by other researchers.
  Replace part of lines 4 and 5 “Oxidative stress which leads to the production of free radicals in the body,” with: Oxidative stress which occurs as a result of imbalance between production and accumulation of free radicals especially reactive oxygen species (ROS),
- **Conclusion** is adequate and reflected the findings of the study.
- I approve that the article be indexed. 

Is the work clearly and accurately presented and does it cite the current literature?

Yes

If applicable, is the statistical analysis and its interpretation appropriate?

Yes

Are all the source data underlying the results available to ensure full reproducibility?

Yes

Is the study design appropriate and is the work technically sound?

Yes

Are the conclusions drawn adequately supported by the results?

Yes

Are sufficient details of methods and analysis provided to allow replication by others?

Yes

Reviewer Expertise:

Phytochemistry and microbiology

I confirm that I have read this submission and believe that I have an appropriate level of expertise to confirm that it is of an acceptable scientific standard.

# Peer review: Reviewer response for version 1

**DOI:** 10.5256/f1000research.27806.r70661

**Published:** 2020-09-17

**Authors:** Abimbola Oloye

**Affiliations:** 1 Department of Veterinary Surgery and Theriogenology, College of Veterinary Medicine, Federal University Of Agriculture Abeokuta, Ogun State, Nigeria

The work prescribed an ethno-medical alternative to treating oxidative stress and perhaps bacterial infections which are largely considered safer, easy to access, and cheaper when compared to most orthodox prescriptions. 

 The wideness of the spread of *H.* *arborescens* especially in southern and eastern Africa makes it fit for consideration as an alternative to some chemically compounded drugs.

 The conclusions drawn about the leaf extracts of this plant by the authors, especially in establishing a relationship between the phenolic content of the extracts and their potencies as antioxidant and antibacterial, were appropriate. However, I wished the authors could give consideration to seasonal variation in concentration statuses of polyphenolics and other bioactive compounds of the plant’s leaves. The month of June during which the study was carried out in the Eastern Cape, South Africa is the winter period, it would be revealing repeating the study in summer (between November and April) and drawing comparisons.

 Studying the effect of blanching was a brilliant one. Indigenes mostly blanch their veggies in the process of making them into edibles or in preparing them as oral medicines for the locals. Blanching, as against chemical extraction, deactivates bioactive compounds hence reduces the therapeutic effects associated with the plant. This has been implied in the study. Interestingly, however, the antibacterial effects of the plant seemed enhanced with blanching. The authors attributed this to enhanced extractive proficiency of water at boiling water temperature. In addition to this, however, I also think the heat-stabile bioactive antibacterial factor should be identified and pinned down.

 The authors should reconcile the justification for the study enumerated in the abstract with the one stated in the introductory section of the manuscript. The assertion that no literature enumerating the phytochemical contents, antioxidative and antibacterial properties of acetone, ethanol and aqueous exist may not be totally correct. There are few studies on these. (See Citations). ^1^^,^^2^ However, they may be correct about the blanched extract. The justification in the introductory section of the main text appears more appropriate.

 The concentrations of saponin and alkaloids determined in the study were said to be enough to elicit the bioactivities associated with the two compounds. Is there any empirical reference that corroborates this assertion especially considering the fact that the authors considered the alkaloid concentration as low?

 A minor correction: The MIC of Ethanol extract against *P. aeruginosa *was 12.5 and this did not fall within the range of 1.563- 3.125 stated in the result. This, therefore, revealed an ethanol extract that had a weak antibacterial effect on *P. aeruginosa*. The authors should take a look at this.

Is the work clearly and accurately presented and does it cite the current literature?

Yes

If applicable, is the statistical analysis and its interpretation appropriate?

Yes

Are all the source data underlying the results available to ensure full reproducibility?

Yes

Is the study design appropriate and is the work technically sound?

Yes

Are the conclusions drawn adequately supported by the results?

Partly

Are sufficient details of methods and analysis provided to allow replication by others?

Yes

Reviewer Expertise:

Reproductive toxicology

I confirm that I have read this submission and believe that I have an appropriate level of expertise to confirm that it is of an acceptable scientific standard.

## References

[1] : Antibacterial, anthelmintic and anti-amoebic activity in South African medicinal plants. *Journal of Ethnopharmacology*.2000;72(1-2) : 10.1016/S0378-8741(00)00269-5 247-263 10.1016/S0378-8741(00)00269-5 10967478

[2] : HETEROMORPHA ARBORESCENS: A REVIEW OF ITS BOTANY, MEDICINAL USES, AND PHARMACOLOGICAL PROPERTIES. *Asian Journal of Pharmaceutical and Clinical Research*.2018;11(11) : 10.22159/ajpcr.2018.v11i11.29108 10.22159/ajpcr.2018.v11i11.29108
